# Microbial lipid-based biodiesel production using wastewater: opportunities and challenges

**DOI:** 10.1186/s40643-025-00897-2

**Published:** 2025-07-04

**Authors:** Ameera Al Shehhi, Yasmine Souissi, Anu Sadasivan Nair, Zeba Usmani, Minaxi Sharma, Nallusamy Sivakumar

**Affiliations:** 1 Department of Biology, College of Science, Sultan Qaboos University, PO Box 36, PC 123 Muscat, Oman; 2 University of Manouba, ISBST, BVBGR-LR11ES31, Biotechpole Sidi Thabet, 2020 Ariana, Tunisia; 3 Department of Engineering, German University of Technology in Oman, Muscat, Oman; 4 Laboratory of Lignin Biochemistry, Department of Chemistry and Biotechnology, Tallinn University of Technology, 12618 Tallinn, Estonia; 5 Department of Food Technology, Akal College of Agriculture, Eternal University, Baru Sahib, Himachal Pradesh 173101 India

**Keywords:** Microbial lipids, Biodiesel, Wastewater, Oleaginous microorganisms, Lipid composition, Biodiesel properties

## Abstract

Rising environmental concerns and fossil fuel depletion necessitate the search for sustainable, alternative energy sources. Biodiesel is emerging as a viable alternative owing to its biodegradability, low toxicity, and reduced greenhouse gas emissions. This review highlights the environmental and economic advantages of biodiesel, with a special emphasis on microbial lipids from wastewater as a promising third-generation feedstock. The novelty of this review is the comprehensive evaluation of wastewater and sewage sludge as low-cost, nutrient-rich substrates for microbial lipid production. In this review, the biodiesel production by direct lipid extraction and microbial conversion routes is discussed. Biodiesel production from different types of industrial wastewaters, such as municipal, dairy, pulp and paper, brewery, and textile wastewater using oleaginous microorganisms is discussed as well. This review revealed that lipid content in different microbes ranged from 27 to 90% when different wastewaters were used as substrates. Further, this review compares the fuel properties of biodiesel derived from plant oils, animal fats, and microbial lipids, their fatty acid profiles, and estimated production costs, and it varies from 0.1 to 0.83 USD/L. In addition, this review also focuses on the biodiesel production from wastewater using microalgae, fungi, and bacteria. Biodiesel with a high cetane number of 64.47 was produced by *Cryptococcus curvatus*. Finally, key challenges in producing biodiesel from wastewater and future prospects are discussed, emphasizing the potential of wastewater as a substrate for sustainable biodiesel production.

**Graphical abstract:**

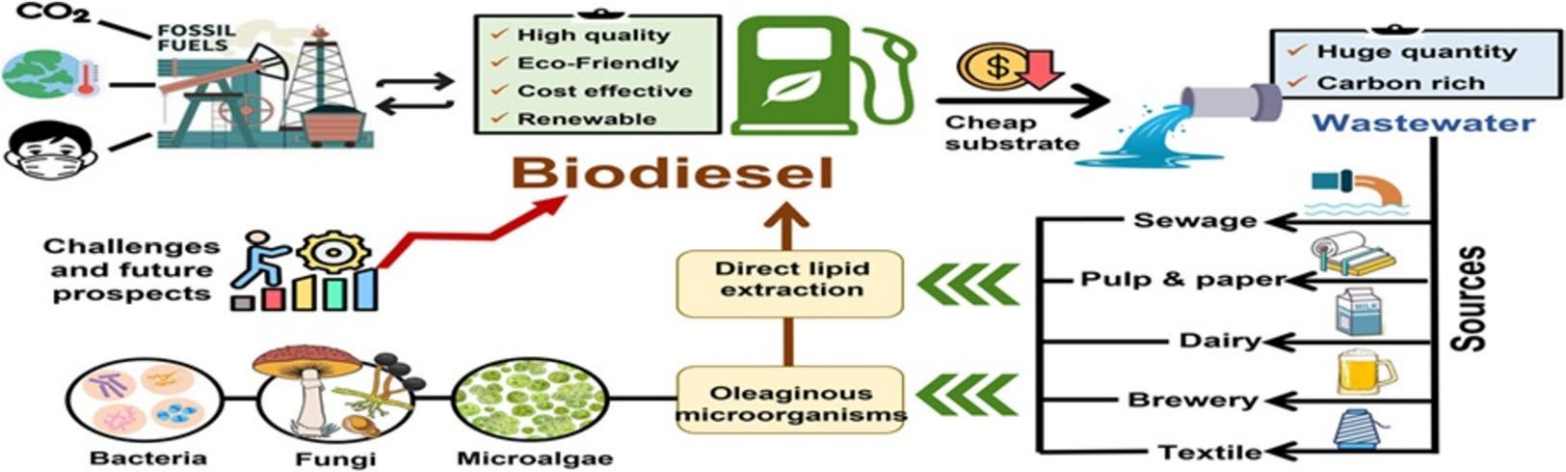

## Introduction

The increasing population and the rapid development in different life sectors demand vast amounts of energy. In 1995, the consumed energy was 8588.9 million metric tons of oil equivalent, which jumped to 13,147.3 million metric tons in 2015 (Dong et al. 2020). Moreover, the total world energy consumption is expected to increase by 28% from 2015 to 2040 (Singh et al. 2024). For a long time, the world has depended mainly on fossil fuels as an energy source, which comprise about 86% of the energy used. The usage of fossil fuels has increased by 1% every year for the last 40 years, reaching 43.33% as a total increase (Singh et al. 2020; Dong et al. 2020). The EIU is expecting that worldwide energy demand will increase by 1.8% in 2024. On the other hand, the demand for fossil fuels will reach high levels, and the demand for renewable energy will rise by 11% (The Economist Intelligence Unit Limited 2024).

The continued consumption of fossil fuels not only leads to the depletion of these sources, which contribute to the global energy crisis, but also leads to several environmental problems, including air pollution. global warming, climate change, acid rain, and ozone layer depletion. The combustion of fossil fuel emits a variety of harmful pollutants, such as carbon dioxide, sulfur, and nitrogen oxides, that negatively affect the environment and human health. Around 5 million deaths annually had been recorded as a result of air pollution, in which fossil fuel burning was the major contributor (Azni et al. 2023; Obada et al. 2024; Meena and Patane 2025). Hence, this issue pushes scientists to seek alternative renewable resources to provide sufficient energy.

Recently, various renewable energy resources have competed with fossil fuels to supply global energy demand while costing less and being friendly to the environment. Many of these resources are abundantly available, such as wind, solar, biomass, etc. (Moosavian et al. 2024). It was reported that the global amount of energy produced from renewable sources in 2023 was approximately 8988.4 terawatt-hours (TWh). Among the different types of renewable energy, hydropower was the main producer, with around 4240.0 TWh; wind energy contributed approximately 2325.3 TWh; solar energy generated about 1641.6 TWh; and other renewable energy, like geothermal and biomass, etc., roughly produced 781.5 TWh. In comparison, fossil fuels (oil, coal, and natural gas) produced 17,957.4 TWh, which was double the amount of energy produced by renewable resources. Moreover, total global consumption showed that 76.5% of consumed energy was generated by fossil fuels. Still, we need more renewable energy to overcome the continuous increase in energy demand and to minimize the dependence on fossil fuels (Ritchie and Roser 2020; International Energy Institute (IEI) 2023).

Choosing the best renewable resource is based on its efficiency, cost, amount of energy produced, and other factors. Biodiesel is a promising source of energy that can be produced from different types of feedstocks, which are classified into different generations. First-generation biodiesel is produced directly from vegetable oils and animal fats. Due to its relatively straightforward production process, it remains the most practical option for commercialization and large-scale production. Nevertheless, its dependency on food crops such as palm and soybean oil raises issues with land use competition, food security, and environmental sustainability (Suhara et al. 2024). In order to overcome the disadvantages of first-generation biodiesel, the second-generation biodiesel utilizes non-edible crops, including agricultural residues, non-edible oil such as sandbox seed oil, and waste oil. However, the economic viability of the second-generation biodiesel is limited due to the need for extensive pretreatment and the complex processing of feedstock. In contrast, third-generation biodiesel, also known as microbial lipid-based biodiesel, offers a more sustainable option. It is produced by using microorganisms such as yeast, algae, and bacteria, which present many advantages by having high growth and productivity, high oil content, no need for agricultural land, and no effect on food supply (Bashir et al. 2022).

Based on the Statistical Review of World Energy, the rate of growth of biodiesel production from 2013 to 2023 was 7.6% per year. However, in 2023, about 960 thousand barrels of oil equivalent per day was the global biodiesel production, which is still lower than global demand (1013 thousand barrels of oil equivalent per day). The main contributors to global output were the European Union (25%), the United States (19%), Indonesia (18%), and Brazil (10%) (EBB 2023). Moreover, the Organization for Economic Co-operation and Development (OECD) reported that by 2031 biodiesel trade is expected to decline from 6.6 bln L to 5.8 bln L, which reflects the low production against the high consumption (OECD 2022). The expected reduction reflects the need for an alternative and sustainable source since biodiesel production mostly relies on vegetable oils.

Microbial lipid-based biodiesel production from waste materials represents a promising avenue for sustainable energy production, promoting both environmental conservation and waste management practices (Suhara et al. 2024). However, using waste materials for energy production is highly advantageous as it will decrease existing environmental waste. Recent research aims to optimize the production process and make waste-based biodiesel an economically viable and widely adopted alternative fuel source. For example, agriculture residues and wastewater sludge could be used to produce biofuels like biodiesel and bioethanol. They are produced in liquid form, making them favorable to supply energy in many sectors like transportation (Saravanan et al. 2018; Nunes et al. 2020). This literature review will focus on biodiesel production using wastewater by oleaginous microorganisms.

## Fossil fuel pollution

**Fig. 1:**
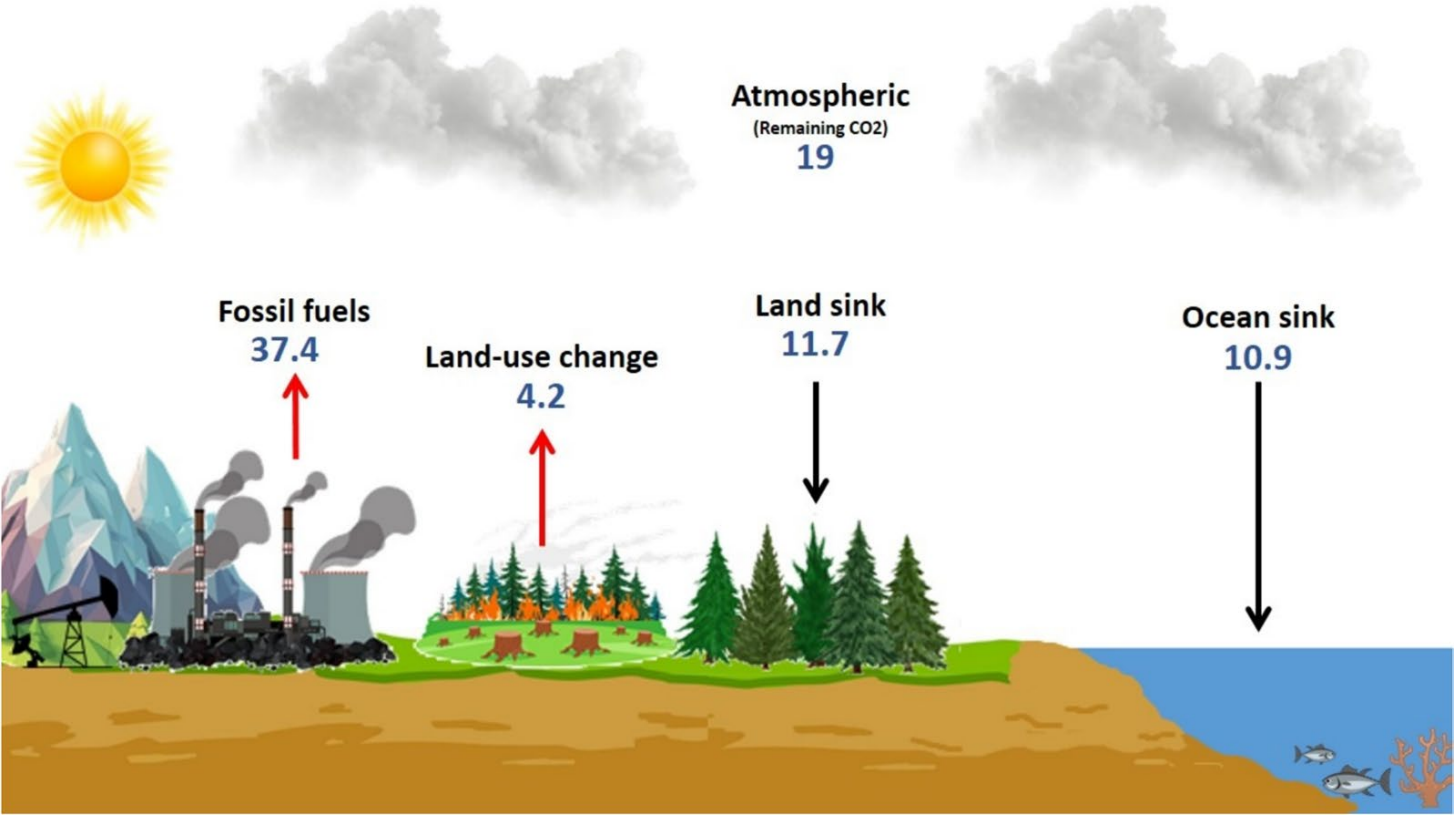
Global CO_2_ emissions and sinks. The values are represented in GtCO_2_/year

Fossil fuels are the main energy source, providing approximately 80% of the total global energy needs (Alalwan et al. 2019). However, continuous efforts have been made to reduce their consumption. This is because of the increasing awareness of their negative impact on the environment. The extraction, transportation, and combustion of fossil fuels have a tremendously harmful impact on the environment (Daudu et al. 2024). Fossil fuels release many pollutants, such as sulfur, nitrogen, and carbon oxides, which cause water contamination, acid rain, and air pollution (Nicoletti et al. 2015; Wang and Azam 2024). The 2024 global carbon budget data showed that the total anthropogenic CO_2_ emission increased by 0.8% in 2024 compared to 2023. Fossil fuels remain the major contributor by releasing about 37.4 GtCO_2_/year (Fig. 1). The imbalance between CO_2_ emissions and natural CO_2_ sinks (land and ocean) has led to the accumulation of around 19 GtCO_2_/year in the atmosphere, accounting for about 30% of the total CO_2_ emitted. Although land uptake (through vegetation, permafrost and soil) and ocean uptake (through marine biota and dissolving to form organic and inorganic carbon compounds) slightly increased in 2024 compared to 2023, they remained insufficient to overcome CO_2_ emissions from fossil fuels. This continuous surplus CO_2_, contributed to various environmental impacts (Pierre et al. 2025).

Approximately 70% of global CO_2_ emissions are due to the combustion of fossil fuels, which causes global warming and climate change. The Sixth Assessment Report (AR6) of the Intergovernmental Panel on Climate Change (IPCC) revealed that the total radiative forcing (RF) from human activities in 2019 was approximately 2.72 W/m^2^, which indicates the extra energy trapped in the Earth and not reflected out to space. This amount was mostly driven by greenhouse gases (GHG), with CO_2_ contributing 1.68 W/m^2^, CH_4_ by 0.48 W/m^2^, N_2_O by 0.17 W/m^2^ and halogenated compounds adding 0.06 W/m^2^. The increase in RF and capturing this amount of energy were the reasons for global warming and climate change, which led to the risk of coastal flooding, heatwaves, agricultural loss, increasing intensity of storms, and health problems (Ebhota and Jen 2020; ISSN 2024). Besides, air pollution has a severe effect on human health. In 2014, the World Health Organization (WHO) reported that air pollution caused the premature deaths of 4–7 million people each year and more than hundreds of millions of illness-suffering people. Infant mortality, allergies, asthma, and cancer are examples of the adverse health impacts of burning fossil fuels and their derivatives (Le et al. 2024). These problems could be completely reduced by using an energy system with zero emissions. Nevertheless, 80% and more of fossil fuel-based energy needs to be converted to zero-emitting energy to avoid an increase in global warming of 1.5 °C by 2030 (Jacobson et al. 2017).

## Importance of biodiesel

The transportation sector is the largest energy consumer which, 93.8% of its energy relays on fossil fuels, specifically diesel, gasoline, and liquefied petroleum gas (Wirawan et al. 2024). However, the tendency to replace them with eco-friendly fuel is increasing. Since the demand for diesel fuel is predicted to increase to 5.7 million barrels per day (Gebremariam and Marchetti 2018), the need for an alternative is necessary to protect the environment. Biodiesel is considered the best choice for replacing petroleum diesel due to its desired properties. Biodiesel is sustainable, renewable, emits fewer pollutants, and is biodegradable (Sujin et al. 2024). Moreover, it has less impact on the environment because it is non-toxic and has no sulfur. Also, it somehow has similar properties to petroleum diesel (Edeh 2020). Consequently, it could be used in most types of diesel engines and equipment without any modification to their manufacturing.

### Environmental benefits

The major advantage of using biodiesel is that it reduces about 90% of the total unburned hydrocarbons during its combustion. Moreover, it emits fewer greenhouse gases, particularly carbon dioxide, in comparison with petroleum diesel. Biodiesel as a fuel will reduce nitrous oxide and sulfur dioxide emissions as it has a lower amount of nitrogen and sulfur. Biodiesel can decompose rapidly, about four times faster than petroleum diesel. According to a study done by Cruz et al. (2019), biodiesel of animal fat origin has more degradation potential as biodiesel is more accessible and can be utilized by soil microorganisms in their metabolic processes. The oxygen content plays an important role in improving the process of biodegradation because the enzymes need oxygen atoms to attack and start dehydrogenation and oxidation reactions in the degradation process. Biodiesel could contaminate natural water through its disposal or usage processes, but it was found that 70–80% of the biodiesel could degrade in one month. However, diesel showed only 18% degradability in the same period. It was found that microbes prefer to degrade biodiesel first and thereafter petroleum diesel (Demirbas 2007; Gupta et al. 2022).

### Biodiesel properties

The quality of biodiesel is assessed by several physicochemical properties, such as cloud point, flash point, viscosity, density, cetane number, and iodine number, based on the ASTM D 6751 and EN 14214 biodiesel standards. These characteristics are crucial for evaluating the ability of biodiesel to replace petroleum diesel. For instance, the cloud point indicates if the biodiesel could be used in both hot and cold areas (Alviso et al. 2020; Nair and Sivakumar 2023). It was reported that biodiesel showed a high cloud point, which means that it can be used in different climatic conditions (Kanakdande et al. 2020). On the other hand, viscosity (the property that shows the fluid resistance to flow over the surface) is the most critical characteristic because it controls the injection process (Alviso et al. 2020). Also, biodiesel showed better combustion quality due to its higher cetane number. The properties of biodiesel derived from different oil sources were analyzed in Table 1 and compared with international biodiesel standards. Cetane Number (CN) is an indicator of ignition quality in diesel engines. According to EN 14214 and ASTM D6751 standards, the minimum required CN for biodiesel is 51 and 47, respectively. Most microbial lipids, jatropha oil, waste cooking oil, and animal fat-based biodiesels exceed these levels, which indicates the excellent combustion feature of these biodiesels. But, the lower CN of sunflower (45.02) and soybean oil (42.52) indicates the need for blending with other fuel.

**Table 1:** Properties of biodiesel derived from plant oils, animal fats, and microbial lipids

Oil source	Cetane no.	Kinematic viscosity (mm^2^/s)	Flash point (°C, min)	Iodine value (g I_2_/100 g)	Density (kg/m^3^)	References
**International standards**						
Europe (EN 14214)	≥ 51	3.5–5.0	≥ 101	≤ 120	860–900	
ASTM D6751	≥ 47	1.9–6.0	≥ 93	–	–	Binhweel et al. (2023)
**Plant oils**						
Sunflower oil	45.02	1.31	–	127.18	870	Ebrahimian et al. (2022)^a^
Soybean oil	42.52	1.29	–	136.27	880	Mora et al. (2024)^a^
Jatropha oil	57.00	4.20	165	–	905	Naveed et al. (2023)
Waste cooking oil	57.38	1.34	–	70.65	850	Suzihaque et al. (2022)^a^
**Animal fats and oils**						
Sheep fat	59.20	4.50	144	–	881	Wang et al. (2025)
Chicken fat	58.40	5.50	183	120	889	Binhweel et al. (2023)
Fish oil	47.00	4.45	177	–	881	
Veal fat	52.34	7.14	–	–	882.5	
**Yeast**						
*Rhodococcus opacus*	59.88	1.54	-	42.29	970	Nair and Sivakumar (2023)
*Rhodotorula genus*	56.99	1.37	–	72.97	860	Pajares et al. (2024)^a^
*Apiotrichum porosum*	56.96	1.39	–	72.38	870	Qian et al. (2021)^a^
*Cryptococcus curvatus*	64.47	1.15	–	69.53	700	Annamalai et al. (2020)^a^
**Micoalgae**						
*Desmodesmus* sp.	58.39	0.96	–	99.55	670	Li et al. (2021)^a^
*Chlorella vulgaris*	49.02	0.27	–	153.92	200	Javed et al. (2022)^a^
*Dunaliella tertiolecta*	49.91	1.28	–	103.53	860	Tizvir et al. (2023)^a^
*Streptomyces rosealbus* and *Asterococcus limneticus*	57.14	1.28	–	68.72	850	Lakshmikandan et al. (2023)

^a^Biodiesel properties were estimated using “Biodiesel Analyzer© Ver. 1.1” based on the fatty acid profiles reported in the referenced studies

Kinematic viscosity plays a major role in fuel injection and atomization. ASTM D6751 allows a wider range of kinematic viscosity (1.9–6.0 mm^2^/s), but EN 14214 is more restrictive (3.5–5.0 mm^2^/s). The biodiesel produced from jatropha oil (4.20 mm^2^/s), sheep fat (4.50 mm^2^/s), chicken fat (5.50 mm^2^/s), and fish oil (4.45 mm^2^/s) are having the values within these limits. However, most microbial sources, such as *C. curvatus* (1.15 mm^2^/s) and *C. vulgaris* (0.27 mm^2^/s) show very low viscosities, suggesting blending with more viscous fuels. Flash point is a parameter indicating the temperature at which fuel vapors ignite. Biodiesel derived from chicken fat biodiesel (183 °C), fish oil (177 °C), and jatropha oil (165 °C) exceeds both EN and ASTM minimums indicating these biodiesels are safer to handle and store.

Plant oil-based biodiesels, especially from soybean (136.27) and sunflower oil (127.18), showed higher iodine values than the EN 14214 limit (≤ 120 g I₂/100g), suggesting poor oxidative stability and a higher risk of rancidity. On the other hand, biodiesel derived from microbial lipids such as *R. opacus* (42.29) and *C. curvatus* (69.53) exhibits lower iodine values, indicating high oxidative resistance and longer shelf life. Moreover, yeasts and microalgae give biodiesel with excellent iodine value which improves the oxidative stability leading to reduction in gum production and engine deposit (Maheshwari et al. 2022). Most biodiesels fall within the acceptable density range of 860–900 kg/m^3^. Particularly, biodiesel from *C. curvatus* (700 kg/m^3^) showed a lower density than the standard and *C. vulgaris* (200 kg/m^3^), show unusually low density, which may be due to experimental variation or require further validation for practical application. Generally, most microbial based biodiesel (third-generation biodiesel) properties meet the standard properties and could be used as an alternative to petroleum-based diesel. The properties could be improved by blending biodiesel derived from different sources or using different types of microorganisms (Brahma et al. 2022).

### Economic benefits

Instead of petroleum diesel, using biodiesel produced from agricultural residues and nonedible oil will balance economic development, agriculture, and the environment (Akram et al. 2022). It could replace diesel, which is the most dominant fuel used globally, including the sectors of agriculture, transport, some industrial applications, and power generation (Rajalingam et al. 2016). It supports the agriculture sector by providing an opportunity for marketing domestic crops. Recently, the biodiesel market has experienced significant growth with an annual rate increase of 6.2%. According to the Biodiesel Market Report 2025, the global biodiesel market was $57.91 billion in 2024, and it is expected to reach $61.52 billion in 2025. Europe holds the largest market share, by over 47.8%, by depending mainly on soybean oil, while the Asia Pacific market is expected to grow much faster in the coming years (IMARC Group 2025).

The raw material is considered the most significant factor, contributing up to 70–95% of the total biodiesel production cost. The 1st generation biodiesel feedstocks are relatively expensive (± 1.01 USD/L) due to the food industry demand for them (Brahma et al. 2022; Feng et al. 2022). However, waste materials such as lignocellulosic biomass and wastewater are used as raw materials to produce biodiesel. On the other hand, the emergence of 3rd generation biodiesel, which is produced from oleaginous microorganisms, is considered a promising shift in the biodiesel economy by minimizing the production cost. As shown in Table 2, the minimum cost for biodiesel production is 0.1 USD/L by using agricultural biomass wastes, while the maximum cost was achieved by using waste cooking oil (0.83 USD/L). Moreover, the data shows that oleaginous yeast like *Trichosporon oleaginosus* could compete with vegetable oil by costing about 0.7 USD/L.

**Table 2:** Estimated production costs of biodiesel from various feedstock types

Feedstock	Yield (v/w)%		Production cost of biodiesel (USD/L)	Year of estimation	References
Sunflower oil		89.1	0.71	2017	Brahma et al. (2022)
Rapeseed oil		37–50	0.75	2020	Brahma et al. (2022)
Palm oil		97.5	0.68	2020	Brahma et al. (2022)
Animal fat		ND	0.44	2019	Esmaeili (2022)
WCO		98.6	0.83	2022	Feng et al. (2022)
Wastewater sludge		ND	0.59	2018	Chen et al. (2018a)
Sewage sludge		93.7	0.46	2023	Usman et al. (2023)
Agricultural biomass wastes: Duroc breed fat oil, *Citrillus lanatus* rind, and Sorghum Bagasse		98.69	0.10	2024	Akwenuke et al. (2024)
*Trichosporon oleaginosus* using municipal sludge and crude glycerol		7.37 g/L	0.72	2020	Kumar et al. (2020a)
Microalgae oil		ND	0.275	2020	Brahma et al. (2022)

*ND* no data available

The inedible feedstock will gain value, and the number of rural manufacturing jobs will increase. Using biodiesel will decrease the money spent on engine maintenance, as it is harmless to the engine. Since it is non-toxic and nonflammable, it needs less care. Biodiesel is very important in terms of energy security in the future, but more concern needs to be raised about reducing its production costs.

## Use of cheaper substrates

Biodiesel is considered an alternative to diesel due to its environmental benefits. However, the major problem with scaling up is the cost. Many factors control the price of biodiesel production, such as the substrate, equipment, pretreatment, production scale, type of biodiesel production, and the possibility of recycling the residual materials. The main factor is the raw materials used in the production (Srikumar et al. 2024). Vegetable oil is used as a feedstock to produce more than 95% of biodiesel. However, the production process is too expensive and cannot compete with petroleum diesel. Furthermore, edible feedstocks are limited in their abundance and interfere with food security. Consequently, many studies were conducted on the use of abundant and cheaper alternative raw materials, for example, non-edible biomass and wastewater. However, they have a low oil yield, but it could be enhanced by using an efficient extraction method (Zabed et al. 2019; Edeh 2020) or using it as a substrate for oleaginous microorganisms. For instance, it was found that the total cost of biodiesel production by using *Rhodosporidium toruloides* DEBB 5533 was US$ 0.76/L which is competitive with vegetable biodiesel origin (US$ 0.81/L) (Soccol et al. 2017). Another study reported that the cost of biodiesel production from lipid in wastewater sludge was 0.59 US$/L, while it costs 0.94 US$/L when only sludge is used to produce biodiesel from the oleaginous microorganism. These results indicate the cheaper production of biodiesel using wastewater sludge. Moreover, the cost could be reduced by increasing the lipid productivity of the raw material and avoiding sterilization, which consumes a high amount of energy. In addition, production costs are affected by the scale of production. It was found that the cost declined by increasing the plant scales. In addition, the use of residuals gained after biodiesel production in some applications, such as fertilizers, will reduce the cost even more (Chen et al. 2018b).

## Wastewater as a source of biodiesel production

Biodiesel is synthesized through the transesterification of animal fats, vegetable oils, or microbial lipids, is a better renewable alternative to petroleum diesel. The high production cost of biodiesel is a major issue for its use in industry (Kumar et al. 2023b). A huge quantity of wastewater is disposed of into the environment from various sources, like domestic (e.g., hotels, houses, and commercial institutions), agricultural activities, industries, and health establishments. For example, palm oil mill alone produces around 250 billion liters of wastewater in the world (Martinez-Burgos et al. 2021). About 75% of the generated wastewater is not treated due to a lack of suitable methods or the high cost of available methods (Sharma et al. 2020). These types of wastewaters differ from each other in their harmful effects on the environment based on their composition, but they all could be used as sources for biodiesel production. To produce value-added products from wastewater, nitrates, phosphates, and other organic chemicals are added as nutrients to enhance microbial growth and fermentation (Chakraborty et al. 2023). The amount and quality of biodiesel will also vary based on the composition of the wastewater used. Reusing and recycling of wastewater can be achieved through bioremediation using various algal species, as they show a high biomass and lipid yield. *Chlamydomonas reinhardtii*, *Monoraphidium braunii*, and *Scenedesmus obliquus* are capable of reducing nitrate, ammonia, phosphate, sulfate, calcium, magnesium, sodium, potassium, and heavy metals such as Zn^2+^, Cu^2+^, Mn^2+^, and Fe^2^ (El-Sheekh et al. 2023).

Using wastewater for biodiesel production has two advantages: protecting the environment from the pollutants existing in the water and providing cheaper material for biodiesel production. Lipids could be obtained in different ways from wastewater, such as through direct extraction and microbial extraction. Although there is enough lipid present in the wastewater, oleaginous microorganisms could produce lipids by using the pollutants as nutrients. Wastewater is rich in nutrients such as organic compounds, nitrogenous compounds, potassium, phosphorous, and some heavy metals, making it suitable for microbial growth and biodiesel production (Girish 2020). As reported in a study done by Sharma et al. (2020), microalgal consortia (MAC1 and MAC2) could utilize domestic wastewater and produce microbial lipids for biodiesel production. In addition, the wastewater sludge contains additional oil or grease oil, which increases the biodiesel production yield (di Bitonto et al. 2024).

## Sewage sludge and wastewater used for biodiesel production

Feedstock selection for biodiesel production is a determinant factor that influences the cost, quality, yield, and applicability of the biodiesel. A wide range of feedstocks are commonly used for biodiesel production, including animal fats, refined vegetable oils, cooking oils, and microbial and algal oils. The feedstocks are classified according to their origins and sources as edible, non-edible, or waste. Nevertheless, to reduce the cost and avoid food-for-fuel conflict, non-edible feedstocks are utilized as the major source for biodiesel production (Praveena et al. 2024). Recently, more attention has been paid to waste-to-wealth biodiesel production as an attractive, environmentally friendly alternative. Waste-to-energy technology advancements enable waste recycling into biodiesel while reducing its disposal and discharge into the environment. Sewage sludge and wastewater represent very suitable feedstock resources for biodiesel production. This section highlights the findings regarding competitive and propitious wastewater feedstock resources for biodiesel production.

### Municipal sewage sludge

Wastewater treatment generates voluminous quantities of sludge that may contribute to environmental hazards if it is not treated and disposed of properly. The quantity of sewage sludge is continuously increasing due to urbanization and industrialization. EU sludge production was estimated at about 9.5 million tons in 2005 and was around 13 million tons in 2020 (Capodaglio and Callegari 2018). More than 6.2 × 10^6^ tons of dried sewage sludge were produced per year from municipal wastewater treatment plants in the USA. The sludge treatment processes consume around 30–80% of the electrical power of the treatment plant and add about 40% to the total greenhouse gas emissions (Guo et al. 2024). Moreover, sludge disposal expenses may represent 30–50% of the total expenditure on wastewater treatment processes (Capodaglio and Callegari 2018).

Sewage sludge is considered an exceedingly available and cheaper feedstock for biodiesel production. A recent review reported that sewage sludge conversion to biodiesel as a sustainable approach to overcome the worldwide problems associated with sewage management (Bora et al. 2020). The economic feasibility associated with the high yield and low cost of sewage sludge is in favour of its use as a valuable feedstock, as 70–80% of the total biodiesel production cost is reported to be raw material cost (Siddiquee and Rohani 2011). In fact, the oil yield from sewage sludge was higher than that from microalgal and soybean oils (Kwon et al. 2012).

**Fig. 2:**
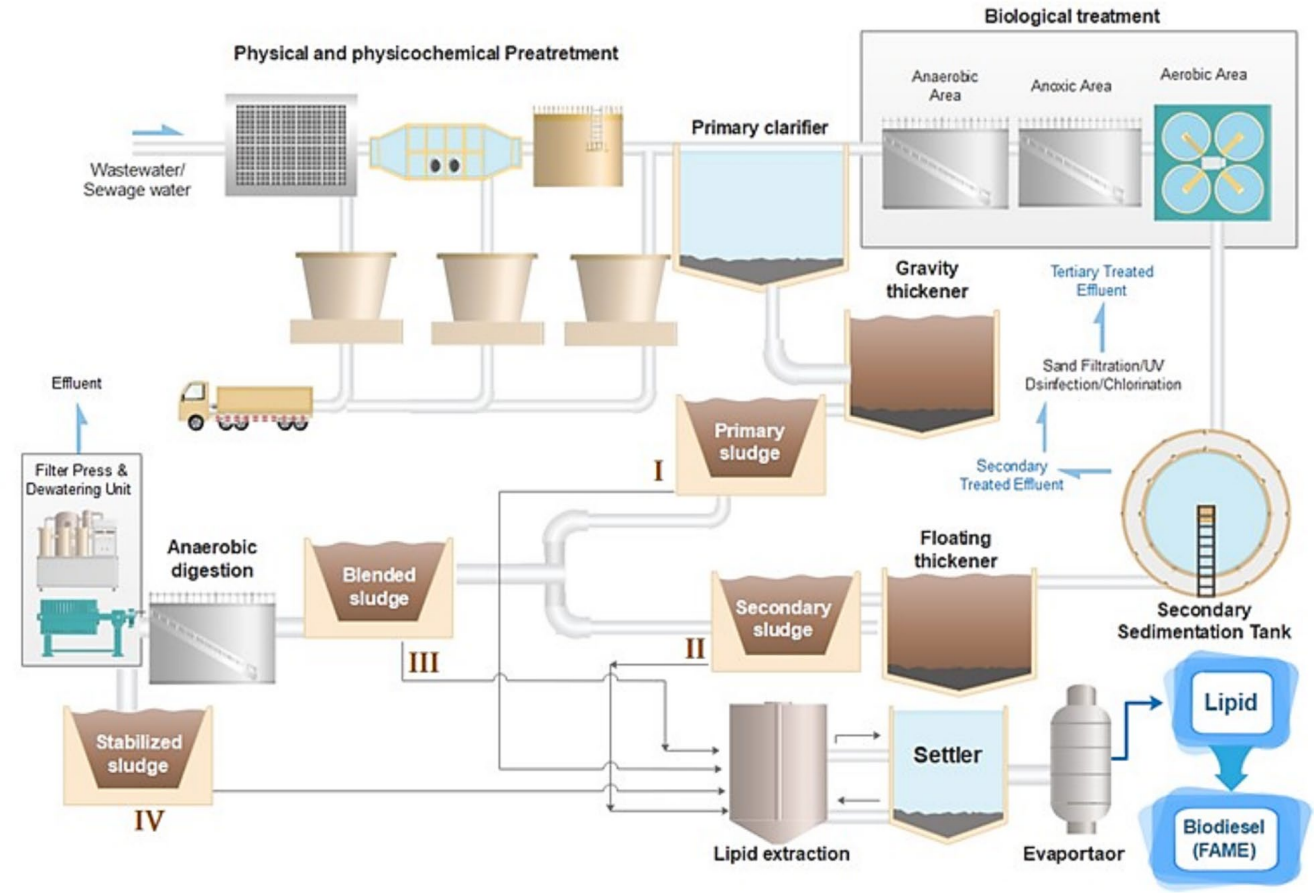
Schematic of a typical wastewater treatment plant with different types of sludge used for biodiesel production

Figure 2 is a schematic representation of a typical municipal wastewater treatment plant integrated with a lipid recovery system for biodiesel production. This figure highlights the different types of sludge that could be considered for lipid extraction and biodiesel production. The wastewater treatment process starts with the physical and physicochemical pretreatment. In this stage, large debris and suspended solids are removed by screening, grit removal, and sedimentation. Then it is followed by mechanical treatment in the primary clarifier to settle down solid and floating grease, which is collected as primary sludge (Bora et al. 2020), whereas the supernatant progresses to biological treatment. The biological treatment consists of anaerobic, anoxic, and aerobic zones. These zones facilitate the nutrient removal and organic matter breakdown through microbial activity. The biologically treated water flows into a secondary sedimentation tank, where secondary sludge is separated. The secondary treated effluent will be subjected to tertiary treatment processes such as sand filtration, ultraviolet (UV) disinfection, or chlorination to meet discharge standards or allow for reuse. The primary sludge undergoes gravity thickening, and the secondary sludge is processed in a floating thickener. Secondary sludge, also called activated sludge, is generated by passing the effluent from the primary clarifier tank through the biological treatment process. Sewage sludge is a complex, heterogeneous mixture containing microorganisms, undigested organic and inorganic materials, and moisture (Tyagi and Lo 2013).

Primary sludge usually contains a combination of organic and inorganic matter, while secondary sludge contains microbial cells and suspended solids. Then these two sludges are blended and processed in parallel by anaerobic digestion and lipid extraction. This anaerobic digestion reduces pathogen content and stabilizes the sludge, thereby making it suitable for safe disposal or use as biosolids. On the other hand, in lipid extraction, the blended sludge is subjected to lipid extraction procedures. These lipids are separated and purified through a settler and evaporator system. These lipids are then transesterified to produce biodiesel, specifically fatty acid methyl esters (FAME). This valorization of wastewater sludge into biodiesel contributes significantly to resource recovery and supports the sustainability of wastewater treatment operations.

The heterotrophic microbial community usually present in the activated sludge is accountable for biological wastewater treatment. These microbial populations use organic compounds present in the wastewater to grow and accumulate lipid droplets as energy and carbon storing compounds (Cea et al. 2015). Some microorganisms can biosynthesize and accumulate triacylglycerol as intracellular oil droplets. Some filamentous bacterial species, including *Mycobacterium*, *Streptomyces*, *Nocardia*, and *Rhodococcus* belonging to the actinomycetes group, were reported as oleaginous bacteria able to biosynthesize triacylglycerol and store more than 20% of their biomass as lipids. The most pertinent bacteria in sludge samples were *Acinetobacter* sp., *Pseudomonas* sp., and *Bacillus* sp. (Cea et al. 2015; Asthana et al. 2024). Extracts of activated sludge obtained from a municipal wastewater treatment plant with a high diversity in the microbial community were investigated for major bacterial lipid storage compounds for both biofuel and oleochemical production. The activated sludge contains bacteria in the phyla *Actinobacteria*, *Bacteroidetes* (class *Flavobacteria* and *Sphingobacteria*), *Firmicutes* (class *Clostridia*), *Proteobacteria* (i.e., *Rhodobacterales* and *Xanthomonadales*), and Verrucomicrobia (class Verrucomicrobiae) (Revellame et al. 2012). The major detected compounds are free fatty acids, free sterols, phospholipids, polyhydroxyalkanoates, wax esters, steryl esters, and triacylglycerides. In addition, diacylglycerides, hydrocarbons, and monoacylglycerides were also detected. The authors indicated that the detected compounds could either be produced by microorganisms or from exogenous sources.

Sewage sludge obtained during municipal wastewater treatment is classified as a solid waste with the code 19 08 05 according to the European Catalogue of Wastes (EEL 47/16-2-2001; Directive 2000/532/EK) (Samolada and Zabaniotou 2014). Municipal sewage sludge discharge faces increasingly stringent environmental requirements due to significant drawbacks influencing air emissions, threatening public health, and contaminating soil and water resources.

Municipal sewage sludge can be converted into a high added-value product, which provides a good alternative substrate for biorefinery. Municipal sewage sludge gained an important attraction as feedstock for biodiesel production as it contains significant quantities of lipids and represents a challenging waste management alternative (Kargbo 2010). Municipal sewage sludge is endowed with a considerable amount of lipids, up to 30 wt.% based on dry sludge (Olkiewicz et al. 2012). The grease and fats are estimated to reach 7–35%TS in the primary sludge and 5–12%TS in the secondary sludge (Tyagi and Lo 2013).

The fatty acid composition of sludge compared with regular biodiesel feedstocks revealed their suitability to produce biodiesel. FAMEs analysis using gas chromatography showed a similar fatty acid profile between the primary and secondary sludge (Mondala et al. 2009; Olkiewicz et al. 2012). Palmitic acid, stearic acid, oleic acid, and linoleic acid were the predominant fatty acids in primary, blended, secondary, and stabilized sludge (Olkiewicz et al. 2012). The suitability of secondary municipal sludge as a promising biodiesel feedstock was also confirmed by the presence of oleic, palmitic, myristic, stearic, lauric, palmitoleic, and linoleic acid methyl esters in the biodiesel profile (Kumar et al. 2016).

The lipid and biodiesel yield from primary, secondary, blended, and stabilized sludge using hexane as an extraction medium were studied. The primary sludge demonstrated the greatest yields, as the amount of extracted lipids was 25.3% and the FAMEs yields obtained were 13.9% (dry wt) (Olkiewicz et al. 2012). These results were confirmed using different pre-treatment methods (ultrasonic and mechanical disintegration), aiming to improve the extraction efficiency (Olkiewicz et al. 2015). The authors also reported that biodiesel production yield was affected by common sludge drying methods, as they lower the yield of lipids and the saponifiable fraction. They described the suitability of direct sequential liquid–liquid extraction using hexane as an interesting alternative associated with previous sludge acidification, which improves lipid and biodiesel yields. After three stages, they were able to recover 91% of the lipid from primary sludge (Olkiewicz et al. 2014).

Primary and secondary municipal wastewater sludge were investigated as a lipid feedstock for biodiesel production. The maximum FAME yields of 14.5 and 2.5% were obtained from primary and secondary sludge, respectively (Zarandi et al. 2023). However, it was found that more than 2,500,000 tons of biodiesel could be produced annually from primary sludge alone (di Bitonto et al. 2020).

### Pulp and paper wastewater

The pulp and paper industry represents a serious threat to the ecosystem due to the discharge of various toxic compounds present in its effluent. Moreover, a significant amount of wastewater is usually associated with this activity. In order to produce a ton of paper, approximately an average of 28.66 m^3^ of water is required worldwide and it ranges from to 80 and 150 m^3^ in some countries (Jauhar et al. 2024). When compared to paper, the wastewater from the pulp and paper industry or paper sludge has negligible lignin content and hence no need for pretreatment methods (Al-Azkawi et al. 2019). Some studies examined microbial lipids as biodiesel feedstock using pulp and paper wastewater effluents as resources using oleaginous microorganisms. More attention should be brought to pulp and paper wastewater, as some studies have reported interesting potential for biodiesel production.

*Rhodotorula glutinis* was used as an oleaginous yeast to utilize pulp and paper wastewater and produce remarkable quantities of lipids in the form of triacylglycerols. Nair and Sivakumar reported that optimizing the appropriate time for collecting the accumulated lipids is critical to making the process economically feasible (Nair and Sivakumar 2022). The fatty acid profile of oleaginous yeast showed palmitic, oleic, and linoleic acids analogous to vegetable oils, making it suitable for biodiesel production (Amirsadeghi et al. 2015). The oleaginous yeast *Rhodosporidium kratochvilovae* HIMPA1 was also exploited as a model organism for its potential to utilize pulp and paper industry effluent as a culture medium (Patel et al. 2017a). Large-sized lipid droplets consisting of high quantities of triacylglycerol or neutral lipids accumulate within its cellular compartment. The FAME profile revealed an acceptable ratio of saturated fatty acids: monounsaturated fatty acids: polyunsaturated fatty acids that improve biodiesel properties (cetane number, oxidation stability, cold behavior properties, and viscosity) according to American Society for Testing and Materials D6751-02 and European Committee for Standardization-EN 14214 guidelines. The potential of *Aspergillus awamori* (MTCC11639) to accumulate lipids was reported. Microbial oil production by *A. awamori* from paper mill effluent was also reported. The high content of saturated fatty acids (SFA) in the FAMEs analysis suggested that the produced fungal oil has similar properties to biodiesel (Venkata Subhash and Venkata Mohan 2014).

Microalgae have been recognized as potential producers of biodiesel. Using wastewater as a nutrient medium for microalgal cultivation is a sustainable and cost-effective approach for biodiesel production and a cheaper way of treating wastewater as well (Lee et al. 2023). Pulp and paper mill effluent were reported to be an interesting media for microalgae cultivation enabling the decrease of freshwater and nutrient requirements (Usha et al. 2016) as it is known that microalgae cultivation, requires an abundance of water and nutrients. For example, *Chlorella vulgaris* and *Scenedesmus acuminatus* were capable of utilizing nutrients in liquid digestates from pulp and paper industry wastewater treatment plants (Tao et al. 2017). This study pointed out that growing *S. acuminatus* in pulp and paper mill biosludge digestates produced a high yield of biomass that could be used for biofuel production.

*Cryptococcus vulgaris* was also a promising candidate for pulp wastewater treatment and lipid and carbohydrate accumulation (Daneshvar et al. 2018a). The authors highlighted that the sum of saturated and monounsaturated fatty acid percentages, the preferred fatty acids for producing biodiesel was higher than 50%. In spite of this promising finding, no studies were conducted on the production of biodiesel with microalgae cultivated on pulp and paper mill wastewater, which was quite intriguing.

### Dairy wastewater

Developing and implementing technically and economically feasible integrated solutions for the management and valorization of dairy wastewater has become an important issue. Microalgae represent a cost-effective alternative for dairy wastewater treatment, ensuring high efficiency in nutrient removal associated with sustainable biodiesel production. The use of microalgae for coupling dairy wastewater treatment with the production of biomass and by-products is a growing and challenging field of interest. In several studies, biodiesel from microalgae has been considered a suitable alternative to conventional fuels. Life cycle analysis suggested that microalgae as feedstock for biofuel production is identified as one of the major renewable energy sources for sustainable development (Medipally et al. 2015). However, microalgal cultivation requires large amounts of water and nutrients, which raises serious concern. The dairy industry generates from 0.2 to 10 L of effluent per liter of milk processed (Gupta et al. 2024). Moreover, the dairy industry liquid waste stream contains a high organic content with a high proportion of protein, nitrogen, phosphorus, dissolved sugars, and nutrients (Ummalyma and Sukumaran 2014). Therefore, using wastewater as an alternative for minimizing freshwater use and reducing the cost of nutrients seems to constitute an interesting route. Cultivating microalgae in dairy wastewater was investigated in several studies highlighting high pollutant removal efficiency and biomass production (Ummalyma and Sukumaran 2014; Ding et al. 2015; Daneshvar et al. 2019).

A recent study reported, in addition to the removal efficiency of total nitrogen, phosphate, and total organic carbon by freshwater microalgae, *Scenedesmus quadricauda*, and marine water microalgae, *Tetraselmis suecica* used for the treatment of dairy wastewater, as well as lipid production abilities, their efficiency toward tetracycline removal via bioadsorption (Daneshvar et al. 2018b). These findings open the doors for the exploitation of microalgae as renewable resources to address sustainable environmental issues while treating dairy wastewater.

Among a variety of microalgae employed in effluent treatment, *Chlorella* is often reported as being well-reputed for its ability to grow in a mixotrophic environment. Moreover, high oil content was reported within *Chlorella* species (Zhang et al. 2023). Quin et al. cultivated *Chlorella* sp. and *Scenedesmus* spp. in monoculture and in consortia in dairy wastewater in order to test their efficiency toward nutrient removal, biomass, and lipid production, as well as their ability to produce renewable biodiesel (Qin et al. 2016). They showed that the maximum total lipid content was observed in the monoalgae *Chlorella* sp. cultivation. However, the maximum lipid productivity was attained in a consortium of *Scenedesmus* spp. and *C. zofingiensis*. They also demonstrated that the lipids produced by the consortium were more worthy of producing biodiesel. *Chlorella pyrenoidosa* for the treatment of dairy wastewater and biodiesel extraction was also investigated. *C. pyrenoidosa* was able to remove about 80–85% and 60–80% of phosphorus and nitrogen, respectively, from the dairy wastewater and has the potential to grow and produce oil that can be used for biodiesel production (Kothari et al. 2012). The authors reported that when influent was used as a growth medium, protein and residual fractions in *Chlorella* biomass were higher than the cultures grown on effluent and growth medium. The biodiesel yield was fourfold higher in influent and threefold higher in effluent than that produced by algal biomass grown in artificial media.

Phycoremediation ability of algal strain *Chlamydomonas polypyrenoideumon* in dairy industry wastewater added to its potential as a source of biodiesel was demonstrated. After 10 days, algal biomass exhibited 42% (w/w) lipid. The FTIR analysis showed that the produced bio-oil was comparable with the bio-oil extracts from other sources (Kothari et al. 2013). Hena et al., isolated and selected algal strains from dairy wastewater based on lipid productivity. Green algae were observed as predominant organisms, followed by cyanobacteria and diatoms, in treated and untreated dairy farm wastewaters. The consortium cultivated in treated wastewater showed a lipid content of 16.89%. This demonstrated a high potential for biodiesel production associated with high and efficient nutrient and COD removal (Hena et al. 2015).

Using dairy farm wastewater, the biodiesel production capability of *Arthrospira platensis* was investigated (Hena et al. 2018). It was demonstrated that this strain accumulated 30.23% lipid content, and the fatty acid profile confirms its suitability as a biodiesel. Comparable total lipid content reached 28.6% for *Spirulina* sp. (*Arthrospira*) grown on digested pig waste when exposed to a lower light flux (Olguín et al. 2001).

*Acutodesmus dimorphus* is a thermotolerant microalgae known for its potential for biofuel production. This strain was successfully exploited in dairy wastewater for biomass generation, phycoremediation and biofuel production. The biomass is composed of 25% lipid and 30% carbohydrate, which could be used to produce biodiesel (Chokshi et al. 2016).

### Brewery wastewater

Beer was reported as the fifth most consumed beverage in the world with an average consumption of 23 L per person per year. The average water utilization in brewing of around 5 to 6 hl/hl of beer is associated with beer production, with two-thirds used in the process and one-third in the cleaning operations. It is to be noticed that the effluent load is almost the same as the water load. This indicates that no water is used to brew beer and ends up as effluent (Fillaudeau et al. 2006). It is estimated that 3 to 10 L of waste effluent is generated for every 1 L of beer produced (Simate et al. 2011). Brewery effluent contains organic compounds, including proteins, phosphates, ammonia, and nitrate, but is also composed of sugars, soluble starch, ethanol, and volatile fatty acids (de J. Raposo et al. 2010). Thus, the brewery industry produces large volumes of water rich in nutrients in favor of microalgal growth. Microalgae consume nitrogen, phosphorus, and other nutrients while growing during the brewery wastewater treatment process (Amenorfenyo et al. 2019). In addition, microalgae release oxygen through their photosynthetic activities, which are utilized by bacteria in wastewater, and they also fix CO_2_ by assimilating HCO_3_ from CO_2_ via respiration.

Combining brewery wastewater treatment with biodiesel production using microalgae could be a promising environmentally friendly alternative with high added-value by-products. A recent review discussed the benefits and challenges associated with microalgae in brewery wastewater treatments that are attracting attention worldwide (Amenorfenyo et al. 2019). Brewery wastewater is considered a sustainable option and a priceless medium for the cultivation of microalgae with high pollution removal efficiencies and high lipid content, suitable for biodiesel production.

The oleaginous species *Scenedesmus obliquus* and *Scenedesmus dimorphus* were reported to contain a high oil content estimated at 11–22/35–55% dry matter and 6–7/16–40% dry matter, respectively (Gouveia and Oliveira 2009). Some studies reported biodiesel production and nutrient removal efficiency using brewery wastewater by *S. obliquus* and *S. dimorphus* (Mata et al. 2012; Lutzu et al. 2016; Marchão et al. 2018; Ferreira et al. 2019). It was reported that a high nutrient removal efficiency (> 99%) was observed with *S. dimorphus* and a high reduction in chemical oxygen demand (65%) (Lutzu et al. 2016). *S. obliquus* showed high removal efficiency by removing 88% N, 30% P, and 71% COD (Ferreira et al. 2019). The biodiesel properties will be enhanced by the composition of long-chain fatty acids, and the degree of unsaturation (Lutzu et al. 2016). The autoxidation is due to the presence of double bonds in the fatty acids (Knothe 2005). Moreover, the positions of allylics with double bonds are especially susceptible to oxidation. The C16–C18 analysis of *S. dimorphus* showed 93.47% fatty acid methyl esters with a relatively high level (67.24%) of unsaturation (Lutzu et al. 2016).

The results obtained for *S. obliquus* present some differences in composition due to the nature of the substrate used for biodiesel production. Mata et al. (2013) used a simulated brewery effluent for cultivation. While Ferreira et al. (2019) used real brewery effluent from a wastewater treatment plant. In fact, Mata et al. showed that the FAME obtained was mainly composed of saturated esters (56.4%), among which the predominant fatty acid was palmitate (C16:0). In their study, regarding the unsaturated esters, the percentage obtained for linolenate (C18:3) is less than the maximum limit of 12% (wt/wt) imposed by the EN 14214:2003 standard. Ferreira et al. reported that microalgal lipids from *S. obliquus* cultivated in real brewery wastewater are mainly composed of unsaturated fatty acids (65%) with a dominance of palmitic (C16:0) and linoleic (C18:2) acids. Linolenic acid (C18:3) estimated at 14.4% (wt/wt) fulfilled EN 14214:2003 standard requirements (Ferreira et al. 2019). These observations support the idea that real brewery wastewater is suitable as feedstock for biodiesel production.

Green algae *Chlorella* sp. is one of the major genera used for wastewater treatment and biodiesel production. *Chlorella vulgaris* efficiency in ensuring both nutrient reduction and sustainable biofuel feedstock production from brewery wastewater treatment was reported in some studies (de J. Raposo et al. 2010; Subramaniyam et al. 2016; Lois-Milevicich et al. 2020). *C. vulgaris* potential for nutrient removal during brewery wastewater effluent treatment was shown to be more efficient with aeration and without light (Choi 2016). Moreover, a high amount of unsaturated fatty acids (83.22%) was observed with aeration in the dark conditions.

Farooq et al. investigated different modes of microalgae cultivation in brewery wastewater to improve the lipid productivity of two strains of *Chlorella* sp. grown (Farooq et al. 2013). The two-stage photoautotrophic–photoheterotrophic cultivation system demonstrated interesting performances for concurrent biodiesel production and brewery wastewater treatment. In addition, the authors showed that adding organic carbon influenced the lipid compositions and improved their content in C18:1, which is more suitable for biodiesel applications.

*Chlorella protothecoides* was also reported as a potential organism for bio-oil production with high biodiesel quality (Kumar et al. 2023a). Anaerobically treated brewery wastewater was used as a low-cost nutrient source to cultivate *C. protothecoides,* with a total nitrogen and phosphorus removal estimated at 96% and 90%, respectively. *C. protothecoides* growth in brewery effluent provides promising alternatives in terms of high lipid contents and improved harvesting efficiency (Darpito et al. 2015). Brewer effluent blended with crude glycerol, a by-product of biodiesel production, was also used to cultivate *C. protothecoides* (Feng et al. 2014). Mixing carbon and nitrogen rich wastes through brewer effluent rich in yeast cell residue known for a high nitrogen content and crude glycerol as a carbon source seems to be an economical and practical alternative for microalgae cultivation and biodiesel production (Feng et al. 2014; Cho and Park 2018).

### Textile wastewater

The textile industry has high water consumption and consequently generates large volumes of wastewater with a high load of contaminants. Textile wastewater effluent is a complex mixture composed of several polluting materials, including organochlorine-based pesticides, heavy metals associated with dyes, surfactants, detergents, and suspended solids (Radha et al. 2009; Malik et al. 2014). Textile effluent is also characterized by a high salinity, high temperature, variable pH, and high chemical oxygen demand (Wang et al. 2016). Depending on the quantity of cloth produced and the type of manufacturing processes used, the discharged effluent from the textile mills range from 1 to 10 million liters per day (Radha et al. 2009). This effluent, also known for its resistance to biodegradation, should be properly treated before discharged into the environment to avoid environmental hazards.

Investigations and the development of textile wastewater treatments are currently receiving much interest. An interesting opportunity may exist in using phycoremediation. As mentioned earlier, microalgae cultivation requires a high amount of water and nutrients, which are provided with textile wastewater. Moreover, textile effluent contains organic dyes, which may constitute a potential carbon source necessary for algal growth. Microalgae are capable of playing a triple function, as they can not only be efficiently used to mitigate textile wastewater but also to fix CO_2_ and produce biodiesel. Different microalgae species including, *C. vulgaris*, *Chlorella pyrenoidosa*, *Spirogyra* sp., *Oscillatoria tenuisin,* and *Scenedesmus* sp., were reported for their ability to remove reactive dyes from textile wastewater (Khalaf 2008; Pathak et al. 2015; Jayakumar et al. 2017; Andrade and Andrade 2018; Fazal et al. 2018). Several mechanisms were described as being involved in the microalgae-based textile wastewater treatment, including biosorption or reductive mechanisms, biocoagulation, bioconversion, and bioaccumulation (Wang et al. 2016; Wu et al. 2017). Considering the presence of toxic dyes and heavy metals, the wastewater grown algae may not be suitable for animal feed use but could be successfully exploited for biodiesel production (Pathak et al. 2015).

The microalga, *Scenedesmus* sp. ISTGA1, was used for the treatment of wastewater and biodiesel production. It was proved to be efficiently utilize nitrate and phosphate, exhibit a high level of heavy metals and organic contaminants reduction, and present a high yield of biomass and lipid content (Tripathi et al. 2019). The authors reported a balanced mixture of saturated and unsaturated fatty acids, mainly (C16 and C18) indicating its suitability for biodiesel production. The textile wastewater treatment by microalga *Chlorella* sp. G23 with simultaneous pollutant removal and lipid production was reported. The addition of phosphate and nitrogen sources proved to improve pollutant removal efficiency as well as FAME production. In fact, after adding urea, the total FAME content was 20.0 ± 3.1%, and the main FAME components were C16:1, C18:2, and C18:3 (Wu et al. 2017).

Lipid production as well as its potential use for biodiesel production from yeasts has been investigated (Galafassi et al. 2012; Tchakouteu et al. 2015; Patel et al. 2017b). Compared with other microorganisms, yeasts are particularly useful for lipid production due to their high multiplication rate, ease of growing on a large scale, less processing time, and endotoxin-free cell biomass (Wang et al. 2012; Ali et al. 2021). Moreover, yeasts have better ability to accumulate intracellular triacylglycerol than other organisms. Table 3 represents microbial lipid content of different microbes using different types of wastewater. A recent research study reported the construction of a multipurpose yeast consortium using *Yarrowia* sp. SSA1642, *Barnettozyma californica* SSA1518, and *Sterigmatomyces halophilus* SSA1511 aiming at lipid production, textile effluent removal, and lignin valorization (Ali et al. 2021). The authors selected these yeasts based on their potential to accumulate intracellular lipids, produce enzymes important for biorefinery, including lipase, cellulases, and hemicellulases, and grow on xylose. The oil obtained from the novel oleaginous consortium was useful for biodiesel production as it contains fewer polyunsaturated fatty acids. The consortium produced about 68.6% of lipid*. R. toluroides* NRRL Y-6987 showed high lipid accumulation in a sweetwater-based medium. In SW_15_ medium, the lipid accumulation is 7.1 × higher than that in SW_1.5_, indicating that the lipid accumulation is induced by lower nutrient conditions and/or a higher C/N ratio (Keita et al. 2024). Different types of wastewaters are tried as substrates to produce lipid through microbial fermentation and have varying yield levels.

**Table 3:** Microbial lipids produced by diverse groups of microbes using different types of wastewaters

Wastewater types	Microbes	Lipid content % (w/w)	References
Scum sludge	ND	27–35	di Bitonto et al. (2020)
Sewage sludge	ND	27.0	Abdulhussein Alsaedi et al. (2022)
Municipal wastewater	*Chlorella pyrenoidosa*	75.6	Tao et al. (2017)
Dairy wastewater	*Chlorella pyrenoidosa*	34.4	Ahmad et al. (2018)
Domestic wastewater	*Chlamydomonas debaryana*	79.0	Ahmad et al. (2018)
Piggery wastewater	*Chlamydomonas. mexicana*	33.0	Girish (2020)
Biogas and wastewater	*Tetraselmis suecica*	41.8	Rafiq et al. (2023)
paper pulp wastewater	*Scenedesmus* sp. and bacteria (aerobic sludge)	22.0	Satiro et al. (2024)
Dairy wastewater	*Chlorella vulgaris*	92.9	Khalaji et al. (2023)
Food-based Brewery industrial wastewater	*Chlorella vulgaris* *Tetraselmis chuii*	33.8 35.0	Usman Shah (2024)
Textile dyeing wastewater	Consortium (*Yarrowia* sp., *Barnettozyma californica* and *Sterigmatomyces halophilus*)	68.6	Ali et al. (2021)
Poultry litter	*Acutodesmus obliquus*	27.4	Musetsho et al. (2021)
Soybean processing wastewater	*Scenedesmus obliquus*	63.9	Shen et al. (2020)
Wastewater from a fish processing plant	*Chlorella vulgaris NIOCCV*	48.0	Trivedi et al. (2019)
Seafood processing wastewater	*Porphyridium cruentum*	27.9	Dewi et al. (2024)
Refined soybean oil wastewater	*Trichosporon fermentans*	43.0	Yu et al. (2018)
Refinery wastewater	*Rhodococcus opacus*	35.6	Paul et al. (2020)

As shown in Table 3 the highest lipid content was achieved using dairy wastewater by *C. vulgaris* giving 92.9% of lipid, while same species in different study give about 33.8% using food-based brewery industrial wastewater. This variation is due to different wastewater used which have different nutrient composition and inhibitor substances. In addition, *C. debaryana* and *C. pyrenoidosa* shows a promising accumulation as well by producing 79.0 and 75.6% of lipid respectively. This is a proof of the strong potential of microbial lipids to be a sustainable feedstock for biodiesel production.

## Biodiesel production

Biodiesel could be produced in several ways, directly or indirectly, from vegetable oils (e.g., palm, corn, coconut, cottonseed, peanut, or sunflower), animal fats, waste cooking oils, wastewater, and oleaginous microorganisms (e.g., fungi, bacteria, and algae). The selection of a proper method depends on many factors, for example, property, cost, required equipment, production methodology, and production yield (Demirbas 2007; Rajalingam et al. 2016).

### Direct lipid extraction

Lipids can be extracted directly from wastewater using chemical methods (Fig. 2) and could be used directly by injecting them into the engine. It was found that the lipid amount extracted from primary, secondary and blended sludge were 27, 9 and 21% respectively. These amounts gave about 19, 4 and 15% biodiesel yield (Olkiewicz et al. 2015). Nevertheless, mixing them with petroleum diesel is preferred to improve quality. Moreover, processing the lipid through different processes like transesterification, pyrolysis (thermal cracking), and microemulsion will give better results. The transesterification process is the best biodiesel production process, among others. The main concept of biodiesel production in the transesterification process is to convert the triacylglycerol in the lipid to biodiesel and glycerol as a byproduct by using alcohol with or without a catalyst (Chanthon et al. 2023; Sharma et al. 2024).

This process was reported to give biodiesel an efficient yield and good quality. Furthermore, its byproduct, glycerol, could be used in some applications. Unfortunately, the produced glycerol was found to be contaminated with some other components (Kumar et al. 2019), which need purification before use. However, the purification process will increase the cost of biodiesel production. Therefore, shifting to using glycerol without purification attracts attention. For example, utilizing it as a substrate to produce more lipids by oleaginous microorganisms, as it is a rich source of carbon (Kumar et al. 2020b).

### Using oleaginous microorganisms

**Fig. 3:**
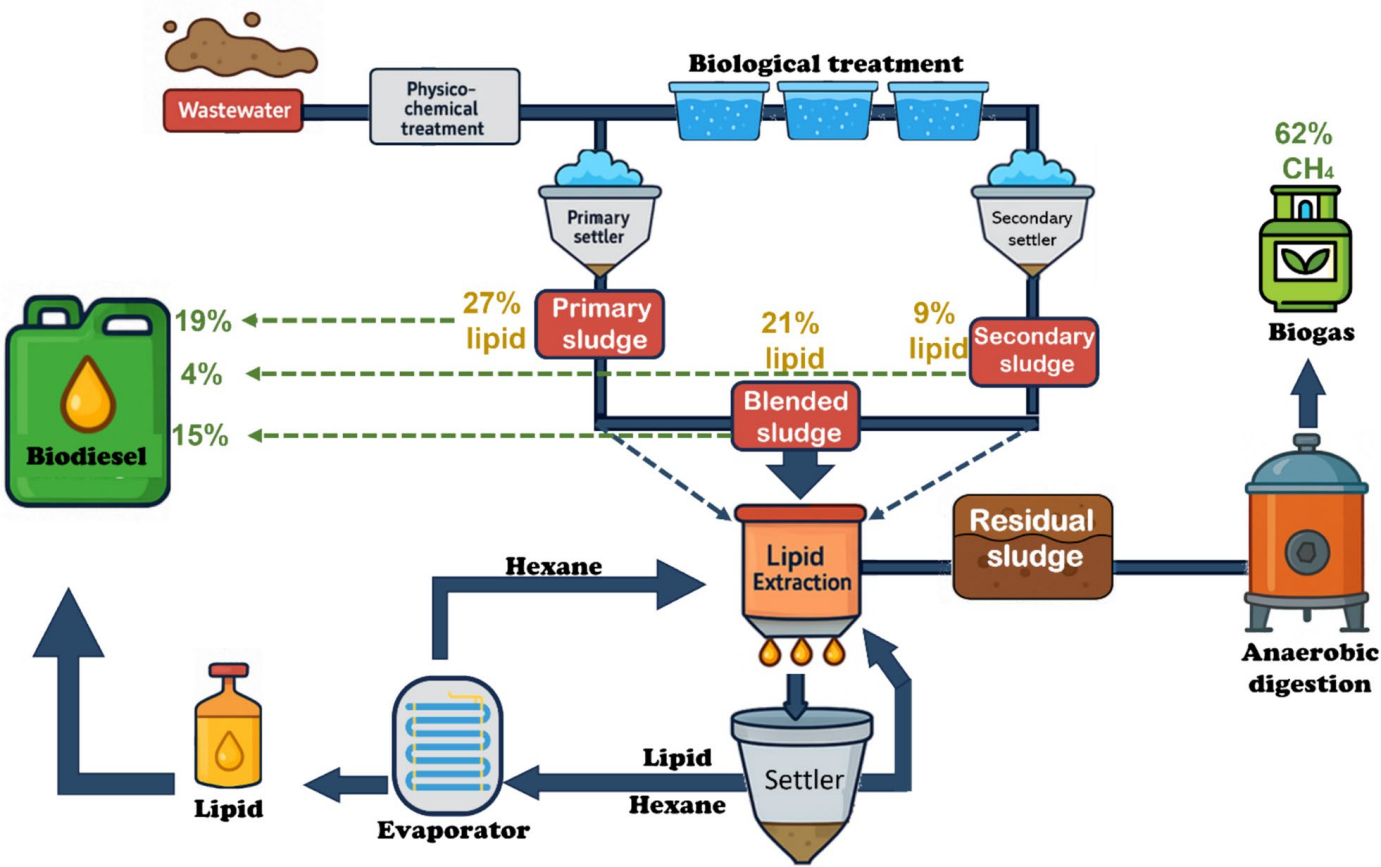
Biodiesel production by direct lipid extraction from wastewater

**Fig. 4:**
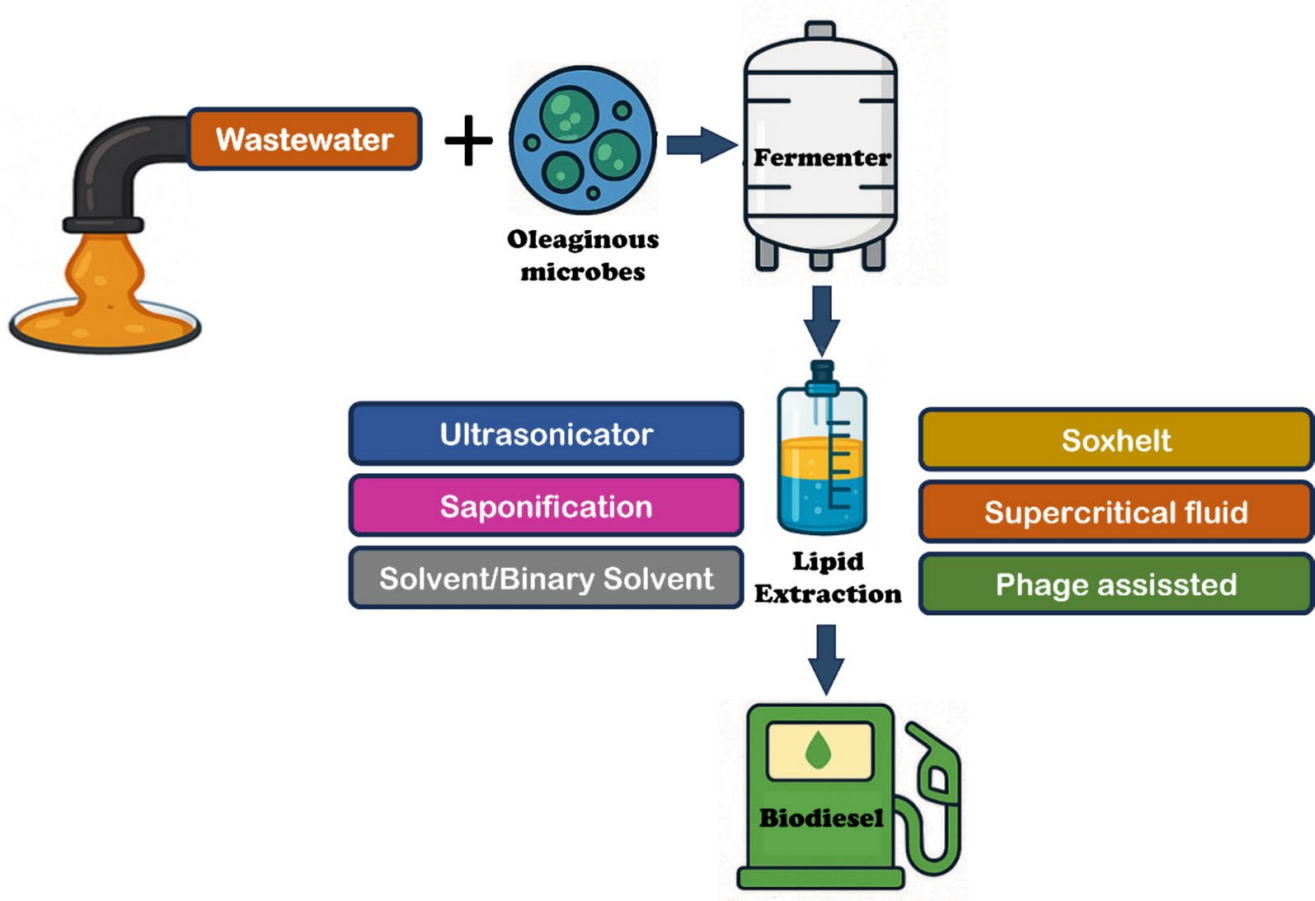
Biodiesel production by oleaginous microorganisms using wastewater as a substrate

Microbial lipids produced by yeasts, fungi, bacteria, and algae are a preferred source of biodiesel production. Microbial lipids are also called single-cell oils, which are edible oils obtained from single-celled microorganisms (Madhariya et al. 2023). Oleaginous microorganisms are capable of synthesizing and storing more than 20% of lipids in their biomass. The high growth rate and the great lipid content (about 80% of dry weight) are the two main advantages of using oleaginous microorganisms in biodiesel production rather than animal fat and oilseed. Furthermore, they could grow independently of climate by providing a suitable environmental condition and reducing land requirements. Their lipid yield could be controlled easily due to the easy modifications in their genetics. Wastewater from different industries can be used as a substrate for microorganisms to produce microbial lipids due to their rich carbon content (Fig. 3). However, microorganisms are cultivated in a fermenter with wastewater contain organic compounds that are converted to intracellular lipids. Next, microbial lipids are recovered from the cell biomass through various methods such as ultrasonication, Soxhlet extraction, saponification, supercritical fluid methods, solvent / binary solvent systems and even phage assisted disruption. To attain more lipid production in oleaginous microorganisms, temperature, salinity, light intensity, and nitrogen starvation are considered the common stress strategies (Singh et al. 2016) (Fig. 4).

There are many different species of bacteria, fungi, yeast, and microalgae that are considered oleaginous microorganisms. Unfortunately, not all of them could be used in biodiesel production. They vary in the amount and type of lipids produced. Oleaginous microorganisms can produce several types of fatty acids, which could be saturated or unsaturated (mono or polyunsaturated fatty acids). The profiles of these fatty acids determined if they are possible to be used in biodiesel production or not (Patel et al. 2020). In addition, oleaginous microorganisms produce and accumulate triacylglycerols (TAGs), which are the focal constituents required for producing biodiesel (Kumar et al. 2019). Consequently, microbial lipid (oil) is considered an alternative resource for biodiesel production. For example, it was reported that microalgae have the ability to produce 9.4 × 10^4^ L/ha of biofuel, which is greater than the amount produced by corn (560 L/ha). Thus, oleaginous microorganisms could solve the challenge of the high production cost of biodiesel. Although the media used in cultivating such microorganisms is expensive, it could be replaced by cheaper renewable sources rich in carbon, mostly obtained from waste (Ullah et al. 2014).

## Types of oleaginous microorganisms used

Three groups of microorganisms are the main producers of lipid, including bacteria (*Rhodobacter capsulatus*, *Flavobacterium hibernum*, *Azonexus caeni*, *Pseudomonas stutzeri*, *Rhodococcus zopfii*), fungi (*Yarrowia lipolytica*, *Cryptococcus vishniaccii*, *Lipomyces starkeyi*, *Rhodotorula glutinis*, *Rhodosporidium toruloides*) and microalgae (*Chlorella vulgaris*, *Chlorella pyrenoidosa*, *Scenedesmus obliquus*, *Thraustochytrium* sp.). It was reported that oleaginous yeasts are superior to microalgae and bacteria in terms of lipid production and growth rate (Qin et al. 2017). Nevertheless, by using wastewater as a substrate, microalgae or a mixture of microalgae and bacteria are revealed to be more appropriate since they are able to remove the pollutants present in the wastewater simultaneously (Zhang et al. 2020). In addition, a co-culture system can use the remaining elements after the lysis of the cells as a substrate for other types of microorganisms (Yen et al. 2015).

### Microalgae

Some microalgae species can accumulate lipids, which makes them a suitable alternative for biodiesel production. Their lipid content can reach up to 90% of their biomass. In addition to their fast growth rate and high lipid content, they can be harvested daily. The lipid accumulation and cell growth depend on various factors such as pH, the intensity of light, the dissolved oxygen level, the carbon dioxide level in sparging gas, nutrients (e.g. phosphorous, nitrogen, iron, and silicon), and the existence of organic carbon sources (Patel et al. 2020).

### Fungi

Oleaginous yeast (single-celled fungi) and filamentous fungi (multicellular fungi) are capable of accumulating lipids in a sufficient amount for biodiesel production. *Rhodosporidium*, *Candida*, *Yarrowia*, *Rhodotorula*, *Cryptococcus*, *Trichosporon*, and *Lipomyces* are examples of yeasts that are characterized by high oil content and a fast growth rate. Some of them can produce lipids up to 80% of their dry cell weight. Oleaginous filamentous fungi have the benefit of producing a special fatty acid profile. γ-linolenic is one of the unique fatty acids that oleaginous filamentous fungi can accumulate in a high amount compared to other oleaginous microorganisms (Patel et al. 2020). However, lipids from fungi are influenced by many factors, such as temperature, pH, agitation, carbon source, nitrogen source, and C/N ratio in broth (Subramaniam et al. 2010). In an experiment performed by Nair et al. (2018), the lipid content of *Cryptococcus curvatus* increased by raising the C/N ratio to 40.

### Bacteria

Oleaginous bacteria produce lipids under certain environmental conditions. Their lipid composition varies, and it depends on bacteria species and carbon sources. Bacteria have great cell growth and high lipid yield using simple cultivation methods, which is considered the main advantage (Nair et al. 2020b). Most bacterial species, such as *Rhodococcus*, *Streptomyces*, *Nocardia*, and *Mycobacterium,* produce lipids in the form of triacylglycerols (TAG) at high concentrations. It was found that *Gordonia* and *Rhodococcus* could produce up to 80% of the lipid content by using media with low nitrogen and high carbon concentrations (Kumar et al. 2019; Patel et al. 2020).

## Composition of lipid production when different wastewater

The composition of the lipid and fatty acid profiles plays an important role in determining the prospect of using them for biodiesel production. Moreover, it controls the quality and properties of the produced biodiesel. Fatty acid profiles vary based on the source of lipid (e.g., microorganisms, vegetable oils, and animal fats). It was reported that the saturated fatty acids, in particular C14, C16, and C18, in addition to the unsaturated fatty acids like C16:1, C16:2, C18:1, C18:2, and C18:3, were the vital fatty acids for gaining biodiesel of good quality (Vilas Bôas and Mendes 2022). The biodiesel production by microorganisms using wastewater as a substrate showed an almost similar fatty acid profile to that of vegetable oil and animal fat substrates. The major components were palmitic acid (C16:0), palmitoleic acid (C16:1), stearic acid (C18:0), oleic acid (C18:1), linoleic acid (C18:2), and linolenic acid (C18:3). This proves that wastewater has the potential to compete as a feedstock for biodiesel production.

However, using different microorganisms and substrates leads to a little variation in the oil profile. As shown in Table 4 the oil profile accumulated in *Tetraselmis indica* using pharmaceutical wastewater missed two types of fatty acids (palmitoleic acid and stearic acid) present in lipids produced by *Chlorella sorokiniana* (Amit et al. 2020). On the other hand, changing the substrate type for the same microorganism could cause a variation in the lipid content (Annamalai et al. 2020). For example, *Cryptococcus curvatus* lipid profile changed by missing palmitoleic acid when spent coffee grounds were used instead of waste office paper as substrates. The quantity of produced lipid and fatty acid profile and their amounts could be varied, as shown in Table 4.

**Table 4:** A comparison of lipid content and fatty acid profiles for different feedstocks for biodiesel production

Species	Substrate	Lipid content (g/L)	Palmitic acid (C16:0)	Palmitoleic acid (C16:1)	Stearic acid (C18:0)	Oleic acid (C18:1)	Linoleic acid (C18:2)	Linolenic acid (C18:3)	References	
*Cryptococcus curvatus*	Spent coffee grounds	36.1	18.9	–	14.0	55.6	8.4	1.5	Titiri et al. (2023)	
	Waste office paper	4.95	3.95	25.69	7.07	50.83	4.25	–	Nair et al. (2020a)	
*Candida tropicalis*	Corn stover	1.09	22.15	2.09	12.77	37.90	6.64	0.54	Luo et al. (2023)	
*Rhodotorula mucilaginosa*	Food industry wastewater	3.25	13.91	–	5.25	73.47	7.37	–	Sundaramahalingam and Sivashanmugam (2023)	
*Cryptococcus laurentii*	Orange peel waste	1.41	22.47	0.26	13.83	46.38	13.63	–	Carota et al. (2020)	
*Rhodosporidium toruloides*	Orange peel waste	2.29	30.94	1.78	4.22	52.02	4.65	–	Carota et al. (2020)	
*Tetraselmis indica*	Pharmaceutical wastewater	1.57	23	–	–	29	7.5	5	Amit et al. (2020)	
*Chlamydomonas globosa*	Agriculture wastewater	0.20	35	–	25	16	12	6	Abd El Fatah et al. (2020)	
*Oscillatoria pseudogeminata*	Agriculture wastewater	0.19	33	–	20	18	15	8	Abd El Fatah et al. (2020)	
*Yarrowia lipolytica*	Palm oil mill wastewater	2.1	23.12	2.57	8.02	43.04	19.14	0.85	Louhasakul et al. (2021)	
*Chlorella sorokiniana*	Wastewater	0.0163	47.61	8.84	10.60	50.39	51.60	2.11	Eladel et al. (2019)	
	Palm oil	30–60	44	0.1	4.5	39.2	10.1	0.4	Kumar et al. (2021)	
Direct extraction of biodiesel	Jatropha oil	38	11.3	–	17.0	12.8	–	47.3	Kumar et al. (2021)	
	Neem	20–30	18	–	15	50	13	–	Kumar et al. (2021)	
	leather tanning waste	62.61	26.8	3.9	14.3	43.3	5.9	2.0	Yuliana et al. (2020)	

For instance, *Chlorella sorokiniana* produced lipid with 47.61% palmitic acid, while lipid produced by *Cryptococcus curvatus* comprised about 18.9% of the same fatty acid. In general, they were similar by having palmitic acid and oleic acid as the major portions of fatty acids in the lipid. This is very crucial for gaining high biodiesel quality. Amit et al. (2020) found that having lipids with more than 50% palmitic acid and oleic acid increased biodiesel quality. Moreover it is obviously that microbial lipid compete vegetable, animal and waste oil sources used to produce biodiesel directly by having similar fatty acid profiles in which palmitic acid and oleic acid were the dominant component. This reflects the ability of microbial lipid to replace the recent sources by having similar fatty acid profiles which indicate similar biodiesel quality.

## Lipid composition and biodiesel properties

Oleaginous microorganisms have an enormous variety of lipid types, such as phospholipids, acyl glycerides, lipoproteins, glycolipids, free fatty acids, hydrocarbons, pigments, and sterols. Each type of lipid is characterized by different physical and chemical properties such as viscosity, solubility, polarity, and cellular location that are important to define their obtainability through extraction (Dong et al. 2016). Oleaginous microorganisms synthesize lipids in two metabolic pathways. In the de novo pathway, lipids are produced due to a limitation in one or more of the nutrients, for example, nitrogen, iron, potassium, phosphorus, magnesium, and sulfur, which are considered essential elements for microorganism proliferation. In such stressful conditions, the cell continues to convert the available carbon to lipids. Triacylglycerols make up around 90% of the produced lipids, and they are composed of 44% unsaturated fatty acids. In addition, they contain free fatty acids, monoacylglycerols, diacylglycerols, and steryl esters, along with a low quantity of polar lipids and sterols. The main fatty acids synthesized are palmitic acid, myristic acid, stearic acid, palmitoleic acid, oleic acid, linoleic acid, and γ-linolenic acid. The *ex-novo* pathway is based on hydrophobic substrates. It is a process in which the microorganism converts the existing free fatty acids into high-value fatty acids (Tzirita et al. 2020). Lipid compositional profiles and properties play an important role in defining biodiesel properties. For example, the degree of unsaturation in the structures of fatty acids influences the quality of biodiesel. The existence of saturated fatty acids in the extracted lipid will give it more thermal efficiency than petroleum diesel. In addition, the presence of monounsaturated fatty acids helps to attain a good balance between cold-flow properties and the cetane number of the biodiesel (Zhang et al. 2020). It was reported that several biodiesel properties, such as cetane number, specific gravity, iodine value, viscosity, and low-temperature performance metrics, associated with the average unsaturation of the FAME profiles. Saturated fatty acids show degradation resistance and consequently increase biodiesel longevity, while unsaturated fatty acids improve cold flow properties. Under high-temperature climatic conditions, saturated fatty acids raise the resistance of biodiesel to oxidation. Therefore, to achieve high biodiesel quality, a suitable ratio of unsaturated and saturated fatty acids should be used. Moreover, the increase in the number of the carbon in FAME leads to an increase in the viscosity of biodiesel, which affects fuel injection. Poor cold flow, lower density, high cloud point, and pour point properties of biodiesel result from the biodiesel composition of long-chain saturated FAME (Hoekman et al. 2012; Talebi et al. 2013).

## Challenges and future prospects

Using biodiesel as an alternate eco-friendly biofuel will reduce the particulate matter released into the atmosphere, which in turn will reduce the impact on global warming. However, the biodiesel does not reduce the emission of NO_2_ due to the presence of a high amount of oxygen (Daud et al. 2015). Nevertheless, biodiesel is a better choice in terms of environmental safety and human health. Production of biodiesel from wastewater is a promising approach to managing these wastes, and it eliminates the competition with food materials and edible oils.

Treating wastewater using a biorefinery approach gained remarkable importance. Biodiesel production using wastewater through a biorefinery approach is a promising way to mitigate environmental pollution. Different types of wastewaters can be utilized as a resource to produce biodiesel by microalgae, fungi and bacteria. Though many studies are showing encouraging results, several challenges still need to be resolved. Priority should be given to the type of wastewater, the quantity of water annually generated, the quality of biodiesel produced from the wastewater, the biomass productivity by the type of microbe used, the lipid accumulating capacity of these microbes, the FAME composition, the harvesting of microbes, energy consumption, and the production cost of the biodiesel.

One of the major challenges in utilizing wastewater for microbial processes is its toxicity, which can directly impair microbial growth. For instance, the presence of heavy metals inhibited the growth of microalgae that has reduced biomass and lipid production (Kwakye Mensah and Ekechukwu 2024). Therefore, wastewater should be analyzed for the presence of toxic substances prior to bioprocess applications. In case of using heavy metal contaminated wastewater, the metal-tolerant microorganisms should be preferred. Using such kinds of microorganisms will benefit the environment by decreasing the pollution and allowing the recovery of water resources beside biodiesel production.

Recently, the intense research in biodiesel production convinced many countries (e.g., the USA, Brazil, and Indonesia) to scale up the production to the industrial level. Still, this transformation depends on the first generation (native crops), which compete for food security. Therefore, many studies have been conducted to use lipid-rich waste (e.g., industrial, agricultural, municipal wastes, and sewage sludge) for biodiesel production. However, the cost of this type of biodiesel production still does not reach a satisfactory level to compete with conventional diesel (Abomohra et al. 2020). There are some suggestions that might be applied to improve and enhance the biodiesel production from wastewater to be more cost-efficient. Co-culture of microorganisms could increase lipid production. An attempt by Suastes-Rivas et al. (2020) to use microalgae-yeast co-culture to produce biodiesel using wastewater revealed that the co-culture technique potentially enhanced the lipid production, biodiesel quality, and consumption of inorganic substances. A similar conclusion of higher lipid and biomass production was achieved by using an alga and bacterium co-culture (*Chlorella sorokiniana* and *Streptomyces thermocarboxydus*) in a study done by Padri et al. (2022), while no significant difference was observed between co-culture and algae monoculture in nutrient removal. Hence, the co-culture technique is a promising method to improve lipid yield and biodiesel quality; however, that depends on the type of microorganisms used and their compatibility in growth.

Researchers should find out the best oleaginous microorganism that gives a very high amount of lipid when cultivated in wastewater. This could be achieved by optimizing microbial lipid producing conditions in the laboratory. But these conditions may not be applicable on a large-scale production as the volume of the treatments differs. In addition, the optimum conditions vary according to the microbes used and the composition of wastewater. Hence, optimizing maximum lipid yield conditions is a tedious and time consuming process. Further, the lipid yielding capacity of oleaginous microorganisms could be improved by genetic engineering techniques in which the lipid producing genes are overexpressed. Moreover, this method offers the chance to generate microbial strains capable of tolerating wastewater toxicity, improved hydrocarbon removal, and increased lipid accumulation (Hassanien et al. 2023). In order to exploit this technique in minimizing biodiesel production cost, more research is needed to explore adding genes that are responsible for increasing the economic value of the bioprocess by inducing additional byproducts producing genes (e.g., pigments and pharmaceuticals with antibacterial and immunomodulatory properties) besides biodiesel production.

In addition, more studies need to be done to determine if any pretreatment for wastewater could enhance production at a lower cost. Though using wastewater as a carbon source for biodiesel production reduces the cost of production to a reasonable extent, the cultivation and harvesting of algae, fungi, and bacteria still retain the reasonable production cost. Similarly, optimizing the conditions to get a higher microbial lipid yield will increase the profit. Naturally, microbes use the available nutrients for many metabolic processes; hence, directing them to produce only lipids is difficult. Although, optimizing the cultivation of the microbes in stressful conditions gives an enhanced accumulation of lipids, it varies among different strains of microorganisms. Moreover, temperature, pH, inoculum concentration, and C/N ratio have a significant influence on biomass and lipid production. Unfortunately, some factors could increase the microbial growth but reduce the lipid accumulation and vice versa. A balanced achievement of biomass and lipid production should be addressed to get a high lipid yield.

Harvesting microbial biomass, especially microalgae, using flocculants is a promising technique used to speed up the sedimentation and increase microbial lipid yield. The challenge is to find a highly effective, cheap, renewable, and eco-friendly flocculant. Chemical flocculants are highly efficient in biomass sedimentation, but they are toxic to the environment. Electro-coagulation–flocculation, magnetically induced membrane vibration system, and bio-flocculants are recently explored alternatives to chemical flocculants (Ali Ijaz Malik et al. 2024).

In addition, novel, cheaper lipid extraction and biodiesel production methodologies should be developed to increase the high lipid yield at a lower cost. Microbial lipids are converted to biodiesel through the transesterification process. To increase the efficiency of the transesterification process and to reduce production costs, a catalyst must be used. However, using traditional catalysts (homogeneous and heterogeneous) has some drawbacks, including instability, cost, soap formation, difficulty in the separation process, and reduction in biodiesel yield (Babadi et al. 2022). These drawbacks demand ongoing research to explore cheap and high-efficiency catalysts. Enzymatic catalysts are promising due to their reusability, renewability, eco-friendliness, and ability to produce high-purity biodiesel with fewer byproducts. Despite these benefits, enzymatic transesterification still faces challenges in large-scale industrial applications. These include sensitivity to process conditions such as temperature, the requirement for specific organic solvents, etc. Further research and process optimization are therefore necessary to fully harness the potential of biodiesel production from microbial lipids using wastewater as a resource.

## Conclusions

Wastewater is a good resource to produce eco-friendly biodiesel. Utilization of wastewater for biodiesel will minimize the effects of wastewater contamination on the environment and the pollution caused by fossil fuel utilization. Wastewater differs in its harmful effect on the environment based on its composition. However, the compositional difference gives an advantage in the lipid composition when used for lipid production using oleaginous microorganisms. The amount and quality of biodiesel produced will also vary based on the composition of the wastewater used.

## Data Availability

Not applicable.

## Abbreviations

FAME: Fatty acid methyl ester
FTIR: Fourier transform infrared spectroscopy
TAG: Triacylglycerols
WWTP: Wastewater treatment plant
